# The effects of hydroxyethyl starch and gelatine on pulmonary cytokine production and oedema formation

**DOI:** 10.1038/s41598-018-23513-0

**Published:** 2018-03-23

**Authors:** Julia Krabbe, Nadine Ruske, Till Braunschweig, Svetlana Kintsler, Jan W. Spillner, Thomas Schröder, Sebastian Kalverkamp, Stephanie Kanzler, Annette D. Rieg, Stefan Uhlig, Christian Martin

**Affiliations:** 1 Institute of Pharmacology and Toxicology, Medical Faculty, RWTH Aachen University, Wendlingweg 2, 52074 Aachen, Germany; 2 Department of Anaesthesiology, Medical Faculty, RWTH Aachen University, Pauwelsstraße 30, 52074 Aachen, Germany; 3 Department of Intensive Care and Intermediate Care, Medical Faculty, RWTH Aachen University, Pauwelsstraße 30, 52074 Aachen, Germany; 4 Institute of Pathology, Medical Faculty, RWTH Aachen University, Pauwelsstraße 30, 52074 Aachen, Germany; 5 Departement of Thoracic and Cardiovascular Surgery, Medical Faculty, RWTH Aachen University, Pauwelsstraße 30, 52074 Aachen, Germany; 6 Department of Surgery, Luisenhospital Aachen, Boxgraben 99, 52064 Aachen, Germany

## Abstract

Recently, side effects of plasma expanders like hydroxyethyl starch and gelatine gained considerable attention. Most studies have focused on the kidneys; lungs remain unconsidered. Isolated mouse lungs were perfused for 4 hours with buffer solutions based on hydroxyethyl starch (HES) 130/0.4, HES 200/0.5 or gelatine and ventilated with low or high pressure under physiological pH and alkalosis. Outcome parameters were cytokine levels and the wet-to-dry ratio. For cytokine release, murine and human PCLS were incubated in three different buffers and time points.In lungs perfused with the gelatine based buffer IL-6, MIP-2 and KC increased when ventilated with high pressure. Wet-to-dry ratios increased stronger in lungs perfused with gelatine - compared to HES 130/0.4. Alkalotic perfusion resulted in higher cytokine levels but normal wet-to-dry ratio. Murine PCLS supernatants showed increased IL-6 and KC when incubated in gelatine based buffer, whereas in human PCLS IL-8 was elevated. In murine IPL HES 130/0.4 has lung protective effects in comparison to gelatine based infusion solutions, especially in the presence of high-pressure ventilation. Gelatine perfusion resulted in increased cytokine production. Our findings suggest that gelatine based solutions may have side effects in patients with lung injury or lung oedema.

## Introduction

Hydroxyethyl starch (HES) and polysuccinated gelatine are frequently used as volume expanders for patients during surgery and in intensive care, as well as a priming fluid during cardiovascular surgery including cardiopulmonary bypass^1–3^. HES is commonly classified by its mean molecular weight and its molar substitution^4^: Hydroxyethyl starch 130/0.4 was developed with a lower mean molecular weight than hydroxyethyl starch 200/0.5 in order to reduce intravascular retention time and the related adverse effects^4^. More recently, the increased incidence of acute kidney injury with need of renal replacement therapy and a higher overall mortality^5–8^ has led to a black box warning by the US Agency for Food and Drug Administration (FDA)^9^ and to the ban by the European Medicines Agency (EMA)^10^ of HES for critically ill patients, including sepsis and burn injury patients. In contrast to these severe renal side effects, in experimental settings HES 130/0.4, but not HES 200/0.5 had anti-oxidative and anti-inflammatory properties and led to improved ventilation and oxygenation^11,12^.

Gelatine solutions, the currently preferred plasma expanders, consist of urea- or succinylated cross-linked modifications of bovine collagen and with 30–35 kDa their molecular weight is noticeably lower than that of HES^13^. Volume therapy with gelatine may be associated with pro-inflammatory responses resulting in cytokine production^14^ and may provoke anaphylactic reactions^15^. Notably, the cytokine inducing properties of gelatine have been already observed in patients with cardiopulmonary bypass^1^.

Isolated perfused lungs (IPL) allow researchers to study the lungs’ responses in the absence of confounding factors such as blood, the nervous system or other organs. When using IPL, perfusion buffers have to match certain physiological properties such as ionic composition, osmolality, pH buffering capacity and oncotic pressure^16,17^. Perfusate oncotic pressure is usually adjusted by addition of high molecular weight components such as HES or gelatine that should secure a stable preparation and minimise cytokine production and oedema formation. The setting of IPL is similar to the situation after extracorporeal circulation in cardiac surgery with dilution of the blood volume with up to 1.5 liters of gelatine or HES containing fluids^2,18,19^.

In this study, we examined the effects of three different plasma expanders in isolated perfused mouse lungs: solutions based on HES 200/0.5 (HES 200), 130/0.4 (HES 130) or gelatine (GELA). Since hypocapnia and respiratory alkalosis may have effects on pulmonary oedema formation^20^, effects of a perfusate pH > 7.45 were also studied. In order to gain insight into the relevance for human lungs, cytokine production was studied not only in perfused lungs, but also in murine and human precision cut lung slices (PCLS).

## Results

### Isolated perfused mouse lung (IPL)

#### Tidal volume, resistance and perfusion pressure

**Figure 1:**
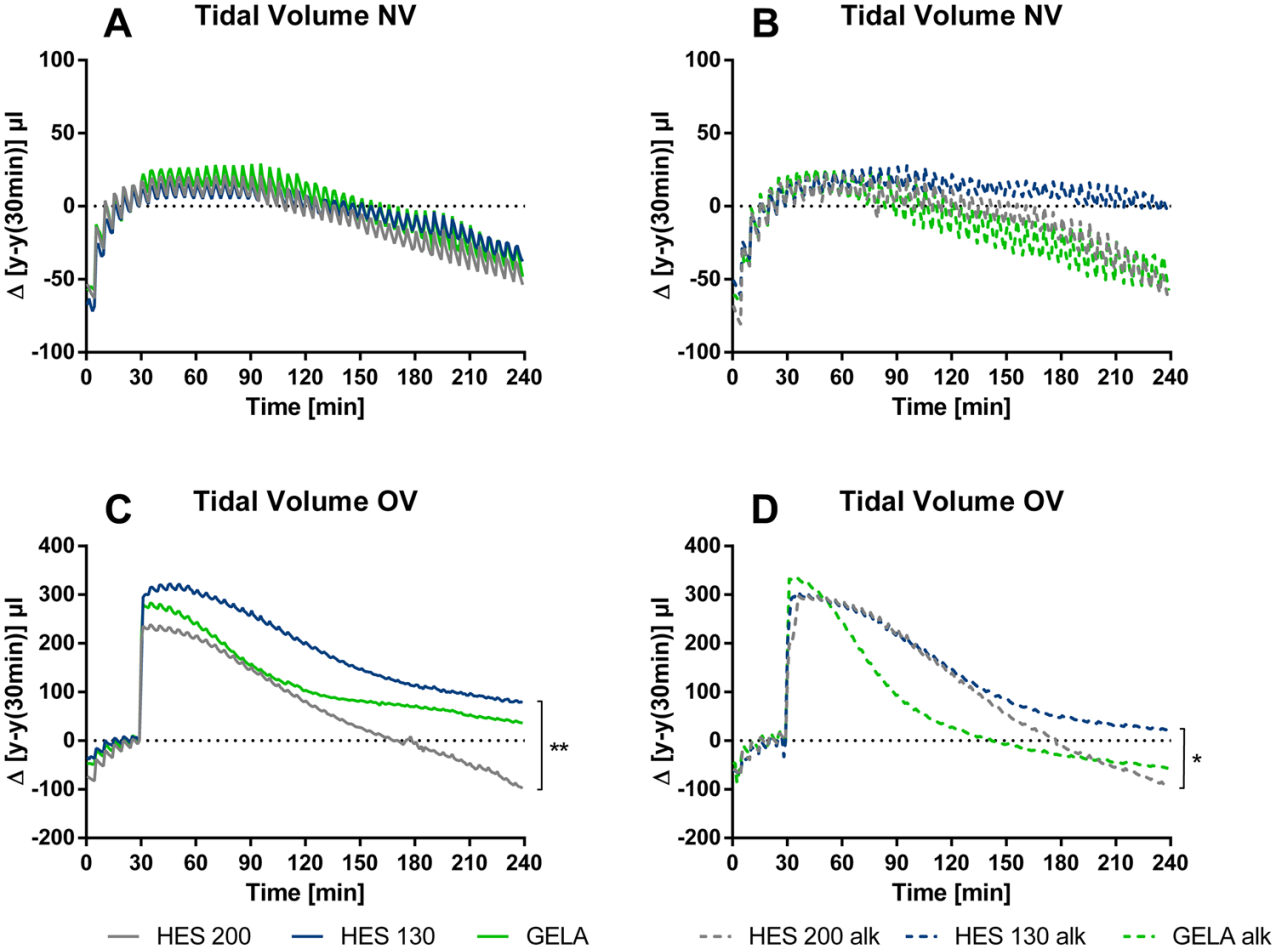
Influence of different buffers on the tidal volume in mouse IPL: Difference of tidal volume over 240 minutes of perfusion and ventilation; overventilation (OV) was initiated after 30 minutes of baseline ventilation. The difference Δ µl is normalised to the initial value at 30 minutes. (**A**) Difference of tidal volume in NV groups under normal pH (mean), (**B**) Difference of tidal volume in NV groups under alkalotic conditions (mean), (**C**) Difference of tidal volume in OV groups under normal pH (mean), (**D**) Difference of tidal volume in OV groups under alkalotic conditions (mean). NV groups: HES 200 n = 6, HES 130 n = 5, GELA n = 6, HES 200 alk = 5, HES 130 alk = 5, GELA alk = 5; OV groups: HES 200: n = 5, HES 130 n = 5, GELA n = 6. HES 200 alk = 6, HES 130 alk = 5, GELA alk = 5. *p < 0.05; **p < 0.01.

All lungs showed an initial increase in tidal volume over time, with normoventilated (NV) groups reaching their plateau after 30–60 minutes (Fig. 1A). In overventilated (OV) groups, the end-inspiratory pressure was increased after 30 minutes of baseline ventilation, leading to a typical increase in tidal volume of about 250 µl (Fig. 1B). Over the 240 minutes of the experiments all lungs decreased in tidal volumes. Comparing the area under the curves (AUCs) of the groups over 240 minutes, no significant differences were found in the NV groups (Fig. 1A and B). However, in OV groups lungs perfused with HES 130 showed significant higher tidal volumes compared to those perfused with HES 200 under normal pH and those perfused with GELA under alkalotic conditions (Fig. 1C and D).

Regarding resistance and perfusion pressure, no significant differences between different buffers, as well as between physiological pH and alkalosis were observed.

#### Perfusate levels of cytokines

All measured cytokine levels showed an increase over time (Suppl. Figure 1), with higher levels in the OV groups compared to the NV groups. However, tumor necrosis factor α (TNFα) levels were always below the detection limit (data not shown), indicating the absence of lipopolysaccharides (LPS) contamination.

**Figure 2:**
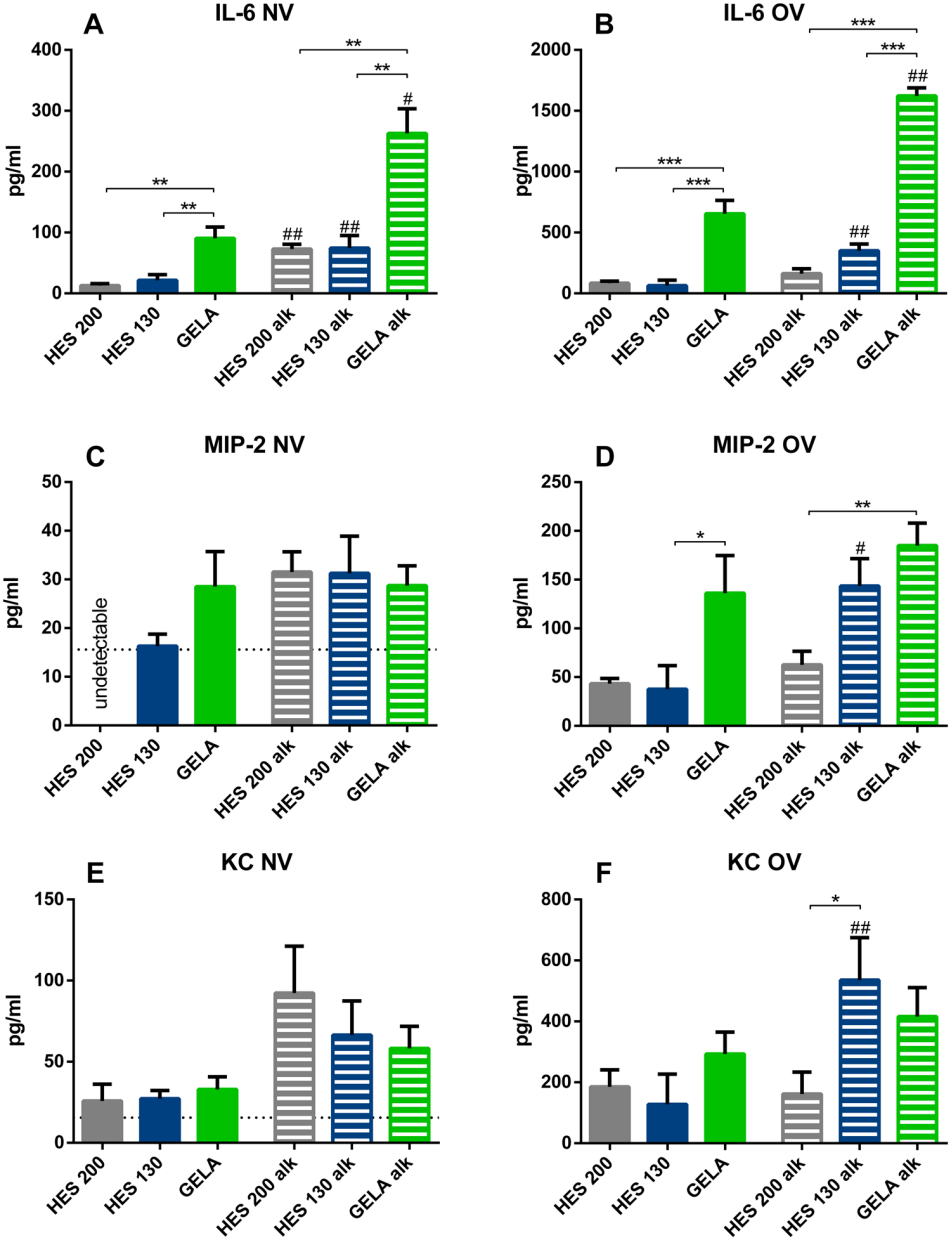
Influence of different buffers and alkalosis on cytokine levels in mouse IPL: Cytokine release of isolated perfused lungs into the perfusate after 240 minutes of perfusion and ventilation. (**A**) IL-6 levels of NV groups (mean ± SEM), (**B**) IL-6 levels of OV groups (mean ± SEM), (**C**) MIP-2 levels of NV groups (mean ± SEM), (**D**) MIP-2 levels of OV groups (mean ± SEM), (**E**) KC levels of NV groups (mean ± SEM), (**F**) KC levels of OV groups (mean ± SEM). NV groups: HES 200 n = 6, HES 130 n = 5, GELA n = 6, HES 200 alk = 5, HES 130 alk = 5, GELA alk = 5; OV groups: HES 200: n = 5, HES 130 n = 5, GELA n = 6. HES 200 alk = 6, HES 130 alk = 5, GELA alk = 5. *p < 0.05, **p < 0.01, ***p < 0.001; ^#^p < 0.05 vs physiological pH, ^##^p < 0.01 vs physiological pH. ^#^Comparison within the same buffer groups for alkalosis or normal pH. The dotted line indicates the MIP-2 detection limit of 15.6 pg/ml.

GELA perfused lungs showed higher interleukin-6 (IL-6) levels after 240 minutes compared to HES 200 and 130 in NV and OV groups under both, physiological and alkalotic conditions (Fig. 2A and B). Macrophage inflammatory protein 2 (MIP-2) levels showed significant differences between GELA and HES 130 in OV at physiological pH and between GELA and HES 200 under alkalotic conditions (Fig. 2D). For keratinocyte-derived chemokine (KC) there was only a significant difference between HES 130 and HES 200 in OV under alkalotic conditions (Fig. 2F).

Alkalosis led to higher IL-6 levels in all three groups of NV lungs (Fig. 2A) and HES 130 and GELA perfused OV lungs (Fig. 2B). For MIP-2 and KC, increased levels were observed for HES 130 perfused lungs in OV groups (Fig. 2D and F).

#### Lung wet-to-dry ratio

**Figure 3:**
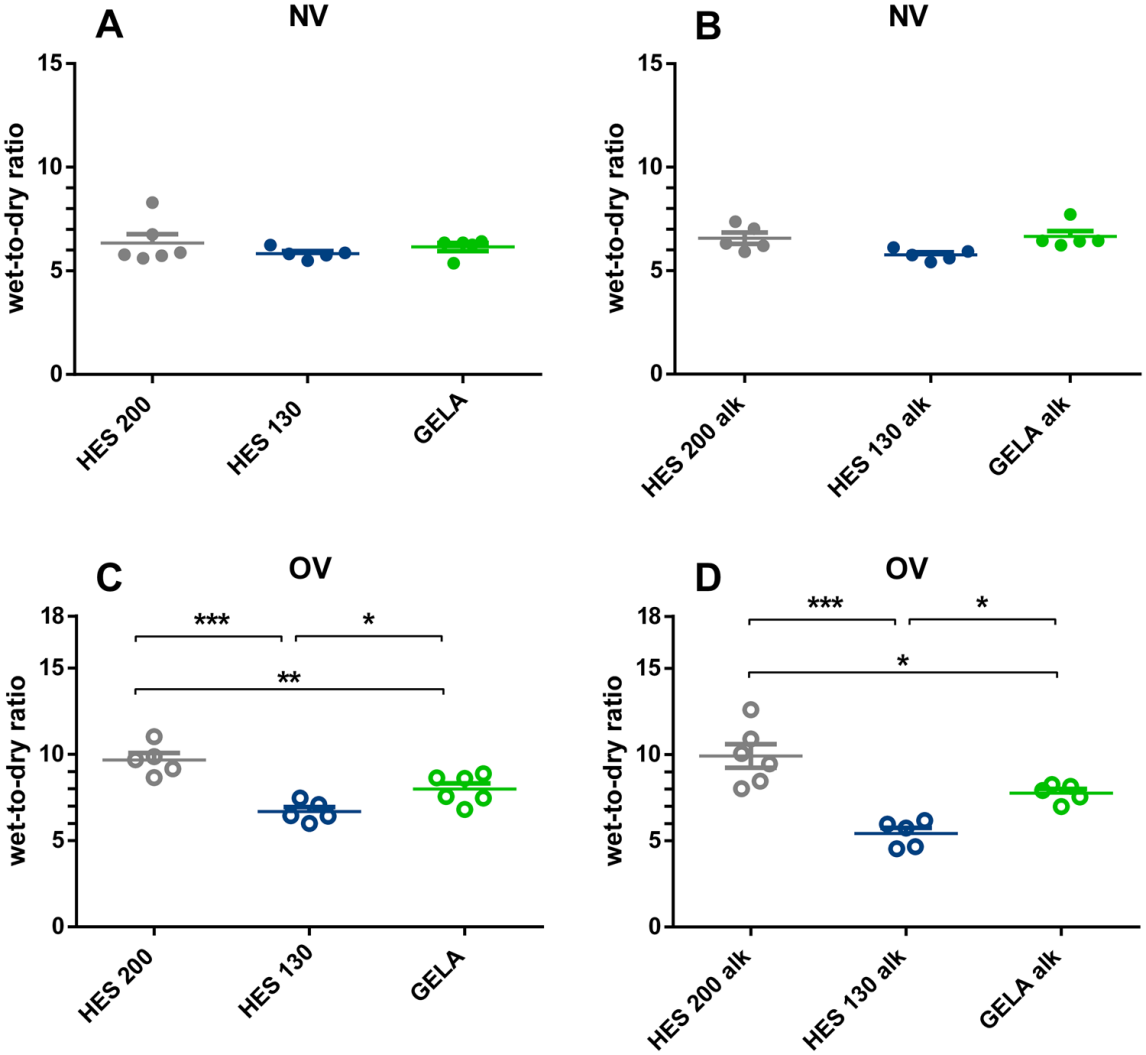
Influence of different buffers on wet-to-dry ratios after 240 minutes of perfusion and ventilation in mouse IPL. (**A**) Wet-to-dry ratios of NV groups under normal pH (mean ± SEM), (**B**) Wet-to-dry ratios of NV groups under alkalotic conditions (mean ± SEM), (**C**) Wet-to-dry ratios of OV groups under normal pH (mean ± SEM), (**D**) Wet-to-dry ratios of OV groups under alkalotic conditions (mean ± SEM). NV groups: HES 200 n = 6, HES 130 n = 5, GELA n = 6, HES 200 alk = 5, HES 130 alk = 5, GELA alk = 5; OV groups: HES 200: n = 5, HES 130 n = 5, GELA n = 6. HES 200 alk = 6, HES 130 alk = 5, GELA alk = 5. *p < 0.05; **p < 0.01; ***p < 0.001.

The wet-to-dry ratio of all groups showed no significant differences in NV groups (Fig. 3A and B). In OV the wet-to-dry ratio of HES 200 was significantly higher than both HES 130 and GELA under normal pH, as well as under alkalotic conditions (Fig. 3C and D). HES 130 buffered lungs showed a decreased wet-to-dry ratio compared to GELA in both OV groups under normal pH and alkalotic conditions (Fig. 3C and D).

#### Osmolality of buffers

The osmolality of HES 200 was 311 mosmol/kg and thus slightly lower than the physiological serum osmolality of healthy mice of 325–330 mosmol/kg^21^, and similar to the osmolality of GELA with 313 mosmol/kg (Table 1). HES 130 solution had a higher osmolality of 330 mosmol/kg, still within the physiological range.

**Table 1:** Basis solutions and recipes of the three buffer solutions: added supplements, ion content and osmolality.

	Volulyte 6% 130/0.4	Gelafundin 4%	HES 200	HES 130	GELA
	g/l	g/l	g/l	g/l	g/l
Hydroxyethyl starch 130/0.4	60	—	—	40	—
Hydroxyethyl starch 200/0.5	—	—	40	—	—
Polysuccinated gelatine	—	40	—	—	30
	**g/l**	**g/l**	**g/l**	**g/l**	**g/l**
Sodium acetate trihydrate	4.63	—	—	3.47	—
Sodium chloride	6.02	3.54	7.2	6.93	5.6
Potassium chloride	0.3	—	0.4	0.45	0.4
Magnesium chloride hexahydrate	0.3	—	—	—	—
Calcium chloride dihydrate	—	—	—	—	—
Calcium nitrate tetrahydrate	—	—	0.1	0.1	0.1
Magnesium sulfate heptahydrate	—	—	0.1	—	0.1
Sodium hydrogen carbonate	—	—	2	2	1.2
Disodium phosphate dodecahydrate	—	—	2.02	—	—
Disodium phosphate dihydrate	—	—	—	—	—
D-(and)-Glucose monohydrate	—	—	2.2	2.2	2.2
Sodium hydrogen sulfate monohydrate	—	—	—	0.06	—
Monosodium phosphate monohydrate	—	—	—	0.78	0.78
Glutathione	—	—	0.001	0.001	0.001
Ultraglutamine I	—	—	0.29	0.29	0.29
			**ml/l**	**ml/l**	**ml/l**
MEM Amino Acids (50x)			20	20	20
MEM Non Essential Amino Acids (100x)			10	10	10
MEM Vitamins (100x)			10	10	10
Water for injections	+	+	—	—	—
pH adjusted with hydrochloric acid or carbon dioxide	+	+	+	+	+
***Ion content***	**mmol/l**	**mmol/l**	**mmol/l**	**mmol/l**	**mmol/l**
Sodium	137	154	158	158	163
Chloride	110	120	129	129	124
Potassium	4	—	5.4	5.4	5.4
Calcium	—	—	0.4	0.4	0.4
Magnesium	1.5	—	0.4	1.0	0.4
Acetate	34	—	—	23	—
Theoretical osmolarity (mosmol/l)	287	274	293	316	293
pH	5.7–6.5	7.1–7.7	adjusted to 7.0	adjusted to 7.0	adjusted to 7.0
Osmolality (mosmol/kg)	278	262	311	330	313

### Precision cut lung slices (PCLS)

#### Cytokine levels

**Figure 4:**
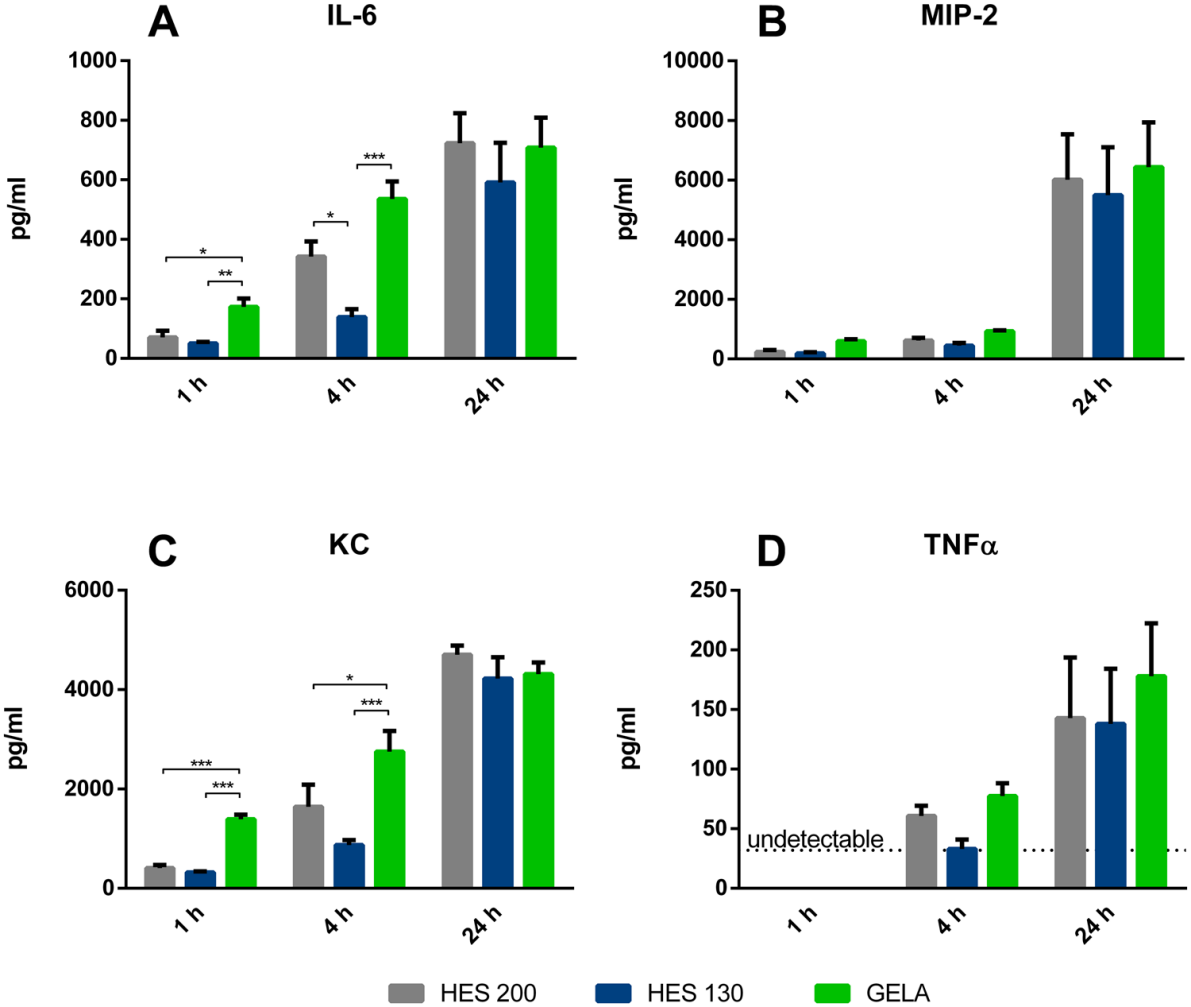
Influence of different buffers on cytokine levels: Cytokine release of murine PCLS into the medium after 1, 4 or 24 hours of incubation (n = 5). (**A**) IL-6 levels after 1, 4 and 24 hours of incubation (mean ± SEM), (**B**) MIP-2 levels after 1, 4 and 24 hours of incubation (mean ± SEM), (**C**) KC levels after 1, 4 and 24 hours of incubation (mean ± SEM), (**D**) TNFα levels after 1, 4 and 24 hours of incubation (mean ± SEM). *p < 0.05; **p < 0.01; ***p < 0.001. The dotted line indicates the TNFα detection limit of 32 pg/ml.

Murine PCLS: GELA incubated slices showed significantly higher cytokine levels in supernatants compared to other buffer groups for IL-6 and KC after one and four hours (Fig. 4A and C). There were no significant differences between groups for MIP-2 and TNFα levels (Fig. 4B and D).

After one hour of incubation, lung slices treated with GELA showed significantly increased IL-6 levels in their supernatants in comparison to HES 200 and HES 130, and in comparison to HES 130 after four hours (Fig. 4A). Regarding KC levels, GELA showed significantly higher levels in comparison to both, HES 200 and HES 130 after one and four hours of incubation (Fig. 4C).

After one hour of incubation no TNFα levels above the detection level could be observed (Fig. 4D).

**Figure 5:**
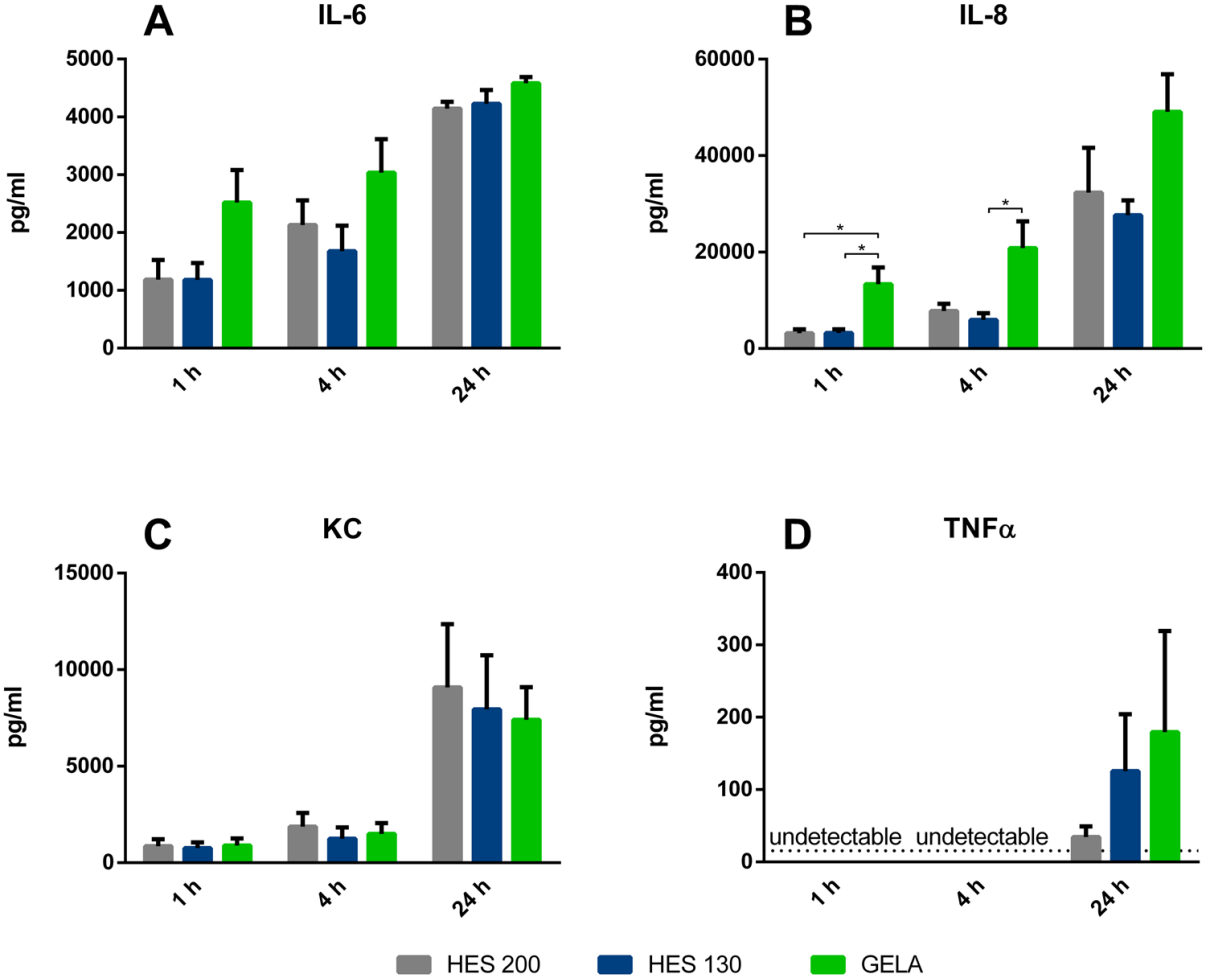
Influence of different buffers on cytokine levels: Cytokine release of human PCLS into the medium after 1, 4 or 24 hours of incubation (n = 5). (**A**) IL-6 levels after 1, 4 and 24 hours of incubation (mean ± SEM), (**B**) IL-8 levels after 1, 4 and 24 hours of incubation (mean ± SEM), (**C**) KC levels after 1, 4 and 24 hours of incubation (mean ± SEM), (**D**) TNFα levels after 1, 4 and 24 hours of incubation (mean ± SEM). *p < 0.05. The dotted line indicates the TNFα detection limit of 15.6 pg/ml.

Human PCLS: Regarding most cytokines, GELA showed the highest cytokine levels, with significantly higher levels of interleukin-8 (IL-8) after 1 hour compared to HES 130 and 200 and after 4 hours compared to HES 130 (Fig. 5B), though similar tendencies and relations between the three tested buffers as in murine PCLS were observed (Fig. 5A–D). Lung slices treated with GELA also presented tendencies of higher IL-6 with no significant differences (Fig. 5A).

## Discussion

The present data suggest that among the tested plasma expanders gelatine and HES 200 in contrast to HES 130 seem to promote lung oedema formation in high pressure ventilated lungs. In addition, buffers containing gelatine tend to be more pro-inflammatory, as suggested especially by increased cytokine release from murine and human tissues.

This is the first study to compare the effects of HES and gelatine-containing solutions on oedema formation and cytokine release in lungs in the absence of blood and other confounding factors. It complements previous *in vivo* studies showing increased cytokine levels after gelatine infusion^1^ and improved pulmonary vascular barrier functions in the presence of HES 130 in adult respiratory distress syndrome (ARDS) or sepsis models^11,22^. IPL is the only *ex vivo* model able to provide reproducible insights in lung oedema formation. The present findings now allow attributing at least some of the effects observed *in vivo* to responses of the lung. Thus, the increased vascular permeability and oedema formation occurred clearly independent of leukocytes and based on the present study one might speculate that these effects are explained by the interaction of GELA with the pulmonary vascular endothelium. This oedema formation might also explain a reduced Horowitz index (PaO_2_/FiO_2)_ ratio in gelatine-infused animals of ARDS (e.g. rabbit lavage model)^11^. Compared to gelatine, HES 130 appears to be tolerated relatively well, especially in lung injury or during high pressure ventilation common in patients with ARDS^23^. The high pressure ventilation in our study induced barotrauma resulting in increased oedema formation and inflammation. The focus of this study was the investigation of the effects of plasma expanders on cytokine production and oedema formation under different circumstances. Plasma expanders are often used in critically ill patients with pathologically altered lungs predisposed for increased lung oedema formation or a preexisting inflammation in the lungs, e.g. patients with acute lung injury and requirement of mechanical ventilation, systemic inflammatory response syndrome (SIRS), ARDS or pneumonia. OV ventilated lungs in our study can grossly mimic these states and give first indications that the tested plasma expanders have similar effects in healthy and injured lungs. Further studies need to determine if the effects are also comparable *in vivo*. We did not study chronically injured lungs corresponding to chronic diseases as pulmonary hypertension, chronic obstructive pulmonary disease (COPD) or emphysema. However, the human PCLS were all received from patients older than 60 years with lung cancer, most of the patients had a confirmed history of smoking and therefore presumable chronic lung injury. Since the cytokine profile as response to the tested plasma expanders did not differ significantly from murine PCLS and IPL, it could be assumed that the investigated responses to HES 130, HES 200 and GELA could be comparable in chronically injured lungs. Further research should address the effects of plasma expanders on chronically injured lungs.

The decreased wet-to-dry ratio indicating decreased lung oedema formation by HES 130, as well as the decreased cytokine production of IL-6, MIP-2 and KC in lung injury by overventilation demonstrate two important effects of HES 130. Both are beneficial for the lung and consequently the lung function, since oedema formation would hinder gas exchange and lung mechanics. Furthermore, an increased cytokine release would initiate or perpetuate inflammation and further impair lung functions.

Clinical studies in human patients investigating the effects of plasma expanders as priming fluids for cardiopulmonary bypass report a peak of cytokine levels 1 to 4 hours after the end of cardiopulmonary bypass, the moment plasma expanders enter the pulmonary circulation^1–3^. Therefore, our experiments may resemble the first phase after separation from cardiopulmonary bypass and introduction of plasma expanders into the circulation and consecutively the pulmonary circulation. Accordingly, in patients plasma levels of HES 130/0.4 were increased the first 4 hours after the infusion^24^. Therefore, relevant effects of plasma expanders would occur in this time frame before elimination via renal excretion. Thus, the investigated time frame in our experiments represents the relevant phase of effects exerted by plasma expanders on the pulmonary circulation.

In addition, the pulmonary oedema formation preventing properties could also originate from the slightly higher osmolality of Volulyte, as well as our HES 130 solution compared to Gelafundin or GELA. However, since the IPL setting lacks abdominal organs including the kidneys, the *in vivo* relevance of our findings requires further study. Due to major differences between lungs and extrapulmonary organs^25^, a differential response to plasma expanders would not be surprising.

In IPL, cytokine release of lung tissue was determined in absence of the major cytokine producing organs, such as thymus, liver and kidneys. Thus, the results cannot easily be generalised and transferred to organisms with an intact body and interacting organ systems. Our buffer in the isolated perfused lungs does not recirculate and therefore no cytokine feedback mechanism inducing further cytokine production exists. Accordingly, Jaecklin and colleagues^26^ observed even higher cytokine levels in a recirculating IPL. Therefore, it can be assumed that cytokine levels would have been even higher in a setting with recirculating perfusion. Since we want to examine the differences between the three plasma expanders, relative changes of cytokines should be sufficient.

Some of the mechanisms seen *in vivo* might also be explained by the higher amounts of pro-inflammatory mediators that were released by the gelatine based buffer in comparison to HES 130. Here, GELA provoked the highest release of IL-6, MIP-2 and KC from perfused lungs, of IL-6 and KC from murine PCLS and of IL-8 from human PCLS. In line with these observations, in rat, rabbit and porcine models HES 130 decreased inflammatory responses, among them nuclear factor ‘kappa-light-chain-enhancer’ of activated B-cells (NF-κB) activation, the expression of cyclooxygenase-2, Intercellular Adhesion Molecule 1 (ICAM-1) and E-selectin, the release of TNF-α, IL-1β and MIP-2, and the extravasation of neutrophils^11,22,27^. Similar observations have also been made in human patients where HES 130 – compared to GELA – reduced pro-inflammatory and increased anti-inflammatory mediators^1^. The present study raises the possibility that some of these anti-inflammatory effects of HES 130 may depend on the lungs, although the mechanisms remain unknown. However, Fujii and colleagues^28^ could also observe anti-inflammatory and endothelial barrier preserving effects of HES 130/0.4 in a rat cardiopulmonary bypass model with detachment of the pulmonary circulation from perfusion and isolated systemic perfusion. The direct comparison of both studies allows the attribution of the effects of HES 130/0.4 to the systemic and pulmonary circulation, as well as connected tissues as site of effects. Notably, those anti-inflammatory effects have not been reported for HES 200 *in vivo*^12,29^. However, in our *ex vivo* experimental set-up migration into the lungs of extrapulmonary cells is not possible. The only cells present in the set-up that can secret cytokines are lung cells like pneumocytes and macrophages and granulocytes present in the lungs at the time point of lung removal. To what extent the cytokine levels and even oedema formation could be altered *in vivo* must be determined in further research.

Human tissue for experiments is very rare and difficult to obtain. Thus, human PCLS can only serve as a “proof of principle” of results obtained in animal experiments. Additionally, the origin of the lung tissue from cancer adjacent tissue has to be considered, although only parts of the lung lobe most distant to the tumor have been selected for this study. This study focuses on the differences between the three plasma expanders and their effects on cytokine production. The comparison of those effects on human lung tissue is unproblematic since all three tested buffers are compared in tissue of the same five human lungs.

In addition, we also examined the effect of increasing alkalosis. Alkalosis resulted in higher IL-6, MIP-2 and KC levels in the perfusate during normal and high tidal volume ventilation, but the major differences between GELA and HES 130 remained. In line with our results, Kimura and colleagues^30^ observed increased cytokine levels (IL-6, TNF-α) in endotoxin-stimulated human whole blood cultures under hypocapnic conditions, i.e. increased pH. While acidosis has been shown to have beneficial rather than detrimental effects attenuating VILI in mouse IPL^31^, alkalosis has not been demonstrated to have any other effects than negative e.g. increased inflammation cause lung damage^32,33^, attenuated hypoxic pulmonary vasoconstriction^34^ and enhanced oedema formation^20,33,35^. Urich and colleagues identified the alkalosis not the hypocapnia as decisive factor for pulmonary oedema formation^20^. Although we did not observe pH-dependent differences in oedema formation (wet-to-dry ratio) or respiratory parameters, our findings suggest that alkalosis should be avoided to limit pulmonary cytokine production and lung injury.

In summary, our results suggest that GELA-based solutions seem to promote increased cytokine release and oedema formation. In the IPL a perfusing buffer has to match certain physiological properties, induce no inflammation and increase the oncotic pressure to avoid lung oedema. For the three tested buffers, HES 130 shows the best properties and can be recommended as perfusing buffer. For human patients the choice of colloid infusion remains difficult and limited, since the indications for hydroxyethyl starch containing infusion solution is restricted to a few emergency situations, e.g. acute haemorrhagic shock not responding to crystalloid infusion^10^. In the light of our results, gelatine could have unfavourable effects in patients with lung injury, lung inflammation or SIRS.

## Methods

### Animals and human lung tissue

Female BALB/c mice (20 ± 3 g) obtained from Charles River (Sulzfeld, Germany) were used as lung donors. They were randomly assigned to one of the groups.

The human lung tissue was collected from patients undergoing lobectomy due to lung cancer. After pathological inspection, cancer free tissue from a peripheral part of the lung was used.

All animal care and experimental procedures were performed according to the rules of the Directive 2010/63/EU of the European Parliament. They were approved by the Landesamt für Natur, Umwelt und Verbraucherschutz, Nordrhein-Westfalen (approval-ID: 84-02.04.2013A146).

The study was approved by the ethics committee (EK 61/09) of the Medical Faculty, RWTH Aachen University. All patients gave written informed consent.

### Agents

Gelafundin 4% was obtained from B. Braun (Melsungen, Germany) and Volulyte 6% from Fresenius Kabi (Bad Homburg, Germany). HES 200 was custom-made by SERAG-Wiessner (Naila, Germany).

Most supplements were obtained from Merck (Darmstadt, Germany), except sodium chloride, potassium chloride, D-(+)-glucose monohydrate, calcium nitrate tetrahydrate, MEM Amino Acids (50x), MEM Non Essential Amino Acids (100x), and MEM Vitamins (100x), which were from Sigma-Aldrich (Steinheim, Germany), and glutathione and ultraglutamine which were purchased from Lonza (Basel, Switzerland). Pentobarbital (Narcoren) was purchased from Merial (Hallbergmoos, Germany), gelatine from porcine skin from Sigma-Aldrich and low melting point agarose from GERBU (Heidelberg, Germany).

### Isolated perfused mouse lung (IPL)

#### IPL preparation

**Figure 6:**
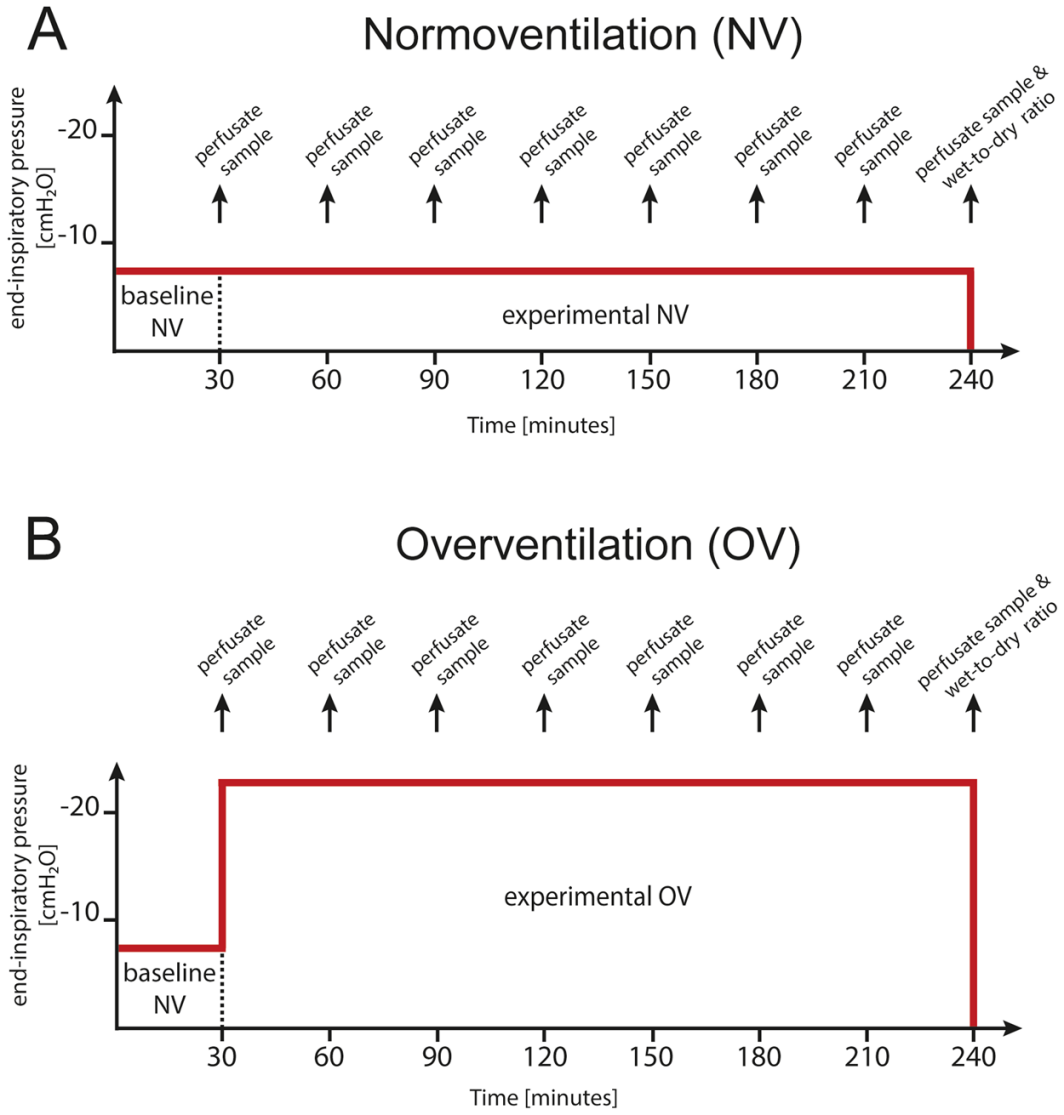
Timeline of mouse IPL experiments: End-inspiratory pressure and sample collection during baseline and experimental ventilation phases. (**A**) Normoventilation (NV) - ventilation was started with an end-inspiratory pressure of −8 cm H_2_O for the first 30 minutes as baseline and the lungs then received the same ventilation for the next 210 minutes. (**B**) Overventilation (OV) - ventilation was started with an end-inspiratory pressure of −8 cm H_2_O for the first 30 minutes as baseline and the lungs then received ventilation with an end-inspiratory pressure of −22.5 cm H_2_O for the next 210 minutes.

IPL were prepared from BALB/c mice as described^36^ and ventilated with a respiratory rate of 90 breaths per minute and an end-inspiratory pressure of −8 cm H_2_O and an end-expiratory pressure of −3 cm H_2_O, resulting in a tidal volume of ~250 µl, for the next 30 minutes as baseline (Fig. 6). Afterwards, mice of the normoventilated groups (NV) received the same ventilation for the next 210 minutes (Fig. 6A), while the overventilated groups (OV) received ventilation with an end-inspiratory pressure of −22.5 cm H_2_O (Fig. 6B) and an end-expiratory pressure of −3 cm H_2_O, resulting in a tidal volume of ~ 500 µl for the same time.

Based on random allocation, HES 130, GELA or HES 200 were used as perfusates with a perfusing speed of 0.5 ml/min resulting in a pulmonary artery perfusion pressure of 0.5 up to 4 cmH_2_O. All buffer solutions were heated to 37 °C before perfusion. All data were transmitted to a computer and analyzed by the Pulmodyn software (Hugo Sachs Elektronik, March Hugstetten, Germany).

#### Composition of perfusate solutions

The established buffer (HES 200) served as reference medium regarding ion content and composition (Table 1). It was initially designed by the recipe of RPMI medium and has been used successfully in many isolated perfused mouse lungs experiments^37,38^.

As the basis for the HES and gelatine buffers we chose two colloid solutions currently available for patient infusion therapy in Germany: Gelafundin 4% and Volulyte 6%. To both solutions multiple ingredients were added to approximate the ion content of HES 200 (Table 1). The colloid solutions had to be diluted to obtain similar ion contents while glutamine, glutathione, as well as vitamin and amino acid supplements were added. After sterile filtration, all solutions were adjusted to a pH of 7.0. To approximate the bicarbonate content, GELA was adjusted with carbon dioxide, while HES 130 and HES 200 were adjusted with hydrochloric acid, already matching the desired bicarbonate content.

#### Perfusion under alkalotic conditions

All buffers were adjusted to a pH of 7.0 before the beginning of the experiments and in the pH controlled groups the perfusing buffer was replaced every 60 minutes with buffer heated to 37 °C and adjusted to a pH of 7.0. This ensured a pH < 7.45 within the one hour intervals, checked by pH measurements at the end of each interval. To mimic conditions of increasing alkalosis, in some groups the pH was only adjusted at the beginning of the experiment and not thereafter. Over the time of the experiment due to carbon dioxide evaporation the pH increased slowly up to a mean pH of 7.75 as determined at the end of the experiments (240 min).

#### Cytokine levels

The investigated cytokines were chosen due to comparability and representation of different pathways of inflammation caused by acute lung injury^39^. IL-8/MIP-2 and KC are important chemokines secreted to facilitate migration of macrophages and neutrophils and their secretion is pivotal for acute lung injury and pulmonary inflammation^40^. Thus, increases in their levels suggest a consecutive leukocyte migration and further inflammation. IL-6 is a typical cytokine of the acute phase response cascade and is produced and secreted in response to injury and infection^41^. TNFα is a typical early inflammatory response to acute lung injury and lung inflammation with further induction of production of radical oxygen species (ROS)^42^. Additionally, TNFα levels serve as technical controls since they reliably indicate bacterial contamination in IPL and PCLS.

Perfusate samples from IPL were analyzed for cytokine levels. The perfusate samples were collected every 30 minutes throughout the IPL experiments. Levels of IL-6, TNFα, KC and MIP-2 were determined by a commercially available enzyme-linked immunosorbent assay (ELISA) according to the manufacturer’s instructions (R&D Systems, Inc., Minneapolis, MN, USA).

#### Lung wet-to-dry ratio

After 240 minutes of ventilation, the left lung was cut at the left hilum and weighed (wet weight). After desiccation at 40 °C for 3 days the dry weight was determined.

#### Osmolality

The osmolality of the tested buffers was measured by freezing-point depression in the Laboratory Diagnostics Centre (LDC) of the University Hospital of RWTH Aachen University. One sample of every buffer was heated up to 37 °C and pH adjusted via hydrochloric acid or carbon dioxide before sending them to testing.

### Precision cut lung slices (PCLS)

#### PCLS from murine and human lung tissue

PCLS from BALB/c mice and human PCLS were prepared as described^36,43^. Briefly, mice were anaesthetised intraperitoneally (160 mg/kg pentobarbital) and the depth of anaesthesia was monitored by missing reflexes. Afterwards, the trachea was cannulated, the abdomen opened and the mouse exsanguinated by incision of the inferior vena cava. Next, the diaphragm and the chest cavity were opened, the pulmonary artery was cannulated and the left ventricle opened via incision. Porcine skin gelatine (6%) was instilled via the pulmonary artery cannula to wash out blood and stabilise the pulmonary circulation. Subsequently, the ventricular incision and pulmonary artery cannula were closed. The murine lungs, as well as the human lung lobes were filled via the trachea or the lobar bronchus with low melting point agarose (final concentration: 1.5%); to solidify the agarose, the lungs or lobes were covered with ice. With murine lungs, both lobes were removed and embedded in low melting point agarose (final concentration: 3%). After cooling, both murine agarose-lobe cylinders and human lung lobes, were cut into 300 mm thick slices with a Krumdieck tissue slicer (Alabama Research & Development, Munford, AL, USA) and incubated at 37 °C in a humid atmosphere in minimal essential medium (MEM) completed with penicillin and streptomycin, containing CaCl_2_ (1.8 mM), MgSO_4_ (0.8 mM), KCl (5.4 mM), NaCl (116.4 mM), glucose (16.7 mM), NaHCO_3_ (26.1 mM), Hepes (25.17 mM), sodium pyruvate, amino acids, vitamins and glutamine. To wash out the agarose and gelatine from the slices, the MEM was changed every half hour during the first 2 hours and every hour during the next 2 hours with following overnight culture. On the following day, the slices were transferred into 24 well plates. All buffers (HES 200, HES 130, GELA) were adjusted to a pH of 7.2–7.3 and 1 ml per well was added. After one, four and 24 hours of incubation at 37 °C in a humid atmosphere, the supernatant was removed and frozen for further analysis.

#### Cytokine levels

Supernatant samples from PCLS were analyzed for cytokine levels. In murine samples levels of IL-6, TNF-α, KC and MIP-2, and in human samples levels of IL-6, TNF-α, KC and interleukin 8 (IL-8) were determined by a commercially available enzyme-linked immunosorbent assay (ELISA) according to the manufacturer’s instructions (R&D Systems, Inc., Minneapolis, MN, USA).

#### Statistics

Data analysis was performed using SAS software v. 9.4 (SAS Institute Inc., Cary, North Carolina, USA), JMP 10 (SAS Institute Inc., Cary, North Carolina, USA), G*Power v. 3.1.9.2 (Düsseldorf, Germany) and GraphPad Prism 5 (GraphPad, La Jolla, USA).

All data are shown as mean ± SEM except in Fig. 1 (mean only) and n indicates the number of animals or lung donors.

Based on the data from Siegl and Uhlig^37^ and own preliminary data, the experiments were planned with a statistical power of 80% and an alpha error of 0.05 (corrected for multiple comparisons) in order to detect differences between the perfusate IL-6 levels greater than 30 pg/ml with a SD of 4.4 (G*Power v. 3.1.9.2, Düsseldorf, Germany), which resulted in a group size of at least n = 5.

For Fig. 1 the area under the curve (AUC) was determined for all individuals. The Shapiro Wilk test was used for verification of normal distribution and the Brown Forsythe test was used to check for equal variances. Analysis was carried out with two-sided unpaired Tukey’s test. For Fig. 3, 4 and 5 the Shapiro Wilk test was used for verification of normal distribution and the Brown Forsythe test was used to check for equal variances. For Fig. 4 and 5, Box Cox transformation was performed to achieve homoscedasticity. Analysis for Fig. 3, 4 and 5 was carried out with two-sided unpaired Tukey’s test.

For Fig. 2 and supplemental Fig. 1 univariate tests were performed using general mixed model analysis (Proc Glimmix; SAS software v9.4) assuming a lognormal distribution for all cytokine data. In case of heteroscedasticity (according to the covtest statement), the df were adjusted by the Kenward-Rogers method. Since the interaction term ‘plasma expander * timepoint’ was always significant with p < 0.05 an increase of all cytokine levels over time can be assumed. Figure 2 depicts the comparison at time point 240 minutes.

### Availability of materials and data

The datasets generated during and/or analysed during the current study are available from the corresponding author on reasonable request.

## Electronic supplementary material

Supplemental Figure 1
