# Supplementary material for: The effects of hydroxyethyl starch and gelatine on pulmonary cytokine production and oedema formation
Source: Sci Rep. 2018 Mar 23;8:5123. doi: 10.1038/s41598-018-23513-0 (PMC5865122; doi:10.1038/s41598-018-23513-0)
Supplement: Supplementary file 1 — Supplemental Figure 1 [file 41598_2018_23513_MOESM1_ESM.pdf]

Supplemental Figure 1

The effects of hydroxyethyl starch and gelatine on pulmonary cytokine production and oedema formation

Julia Krabbe[1/2/3*], Nadine Ruske[1], Till Braunschweig[4], Svetlana Kintsler[4], Jan Spillner[5], Thomas Schröder[6], Sebastian Kalverkamp[5], Stephanie Kanzler[1], Annette D. Rieg[1/2], Stefan Uhlig[1] and Christian Martin[1]

[1]Institute of Pharmacology and Toxicology, Medical Faculty, RWTH Aachen University, Wendlingweg 2, 52074 Aachen, Germany

[2]Department of Anaesthesiology, Medical Faculty, RWTH Aachen University, Pauwelsstraße 30, 52074 Aachen, Germany

[3]Department of Intensive Care and Intermediate Care, Medical Faculty, RWTH Aachen University, Pauwelsstraße 30, 52074 Aachen, Germany

[4]Institute of Pathology, Medical Faculty, RWTH Aachen University, Pauwelsstraße 30, 52074 Aachen, Germany

[5]Departement of Thoracic and Cardiovascular Surgery, Medical Faculty, RWTH Aachen University, Pauwelsstraße 30, 52074 Aachen, Germany

[6]Department of Surgery, Luisenhospital Aachen, Boxgraben 99, 52064 Aachen, Germany

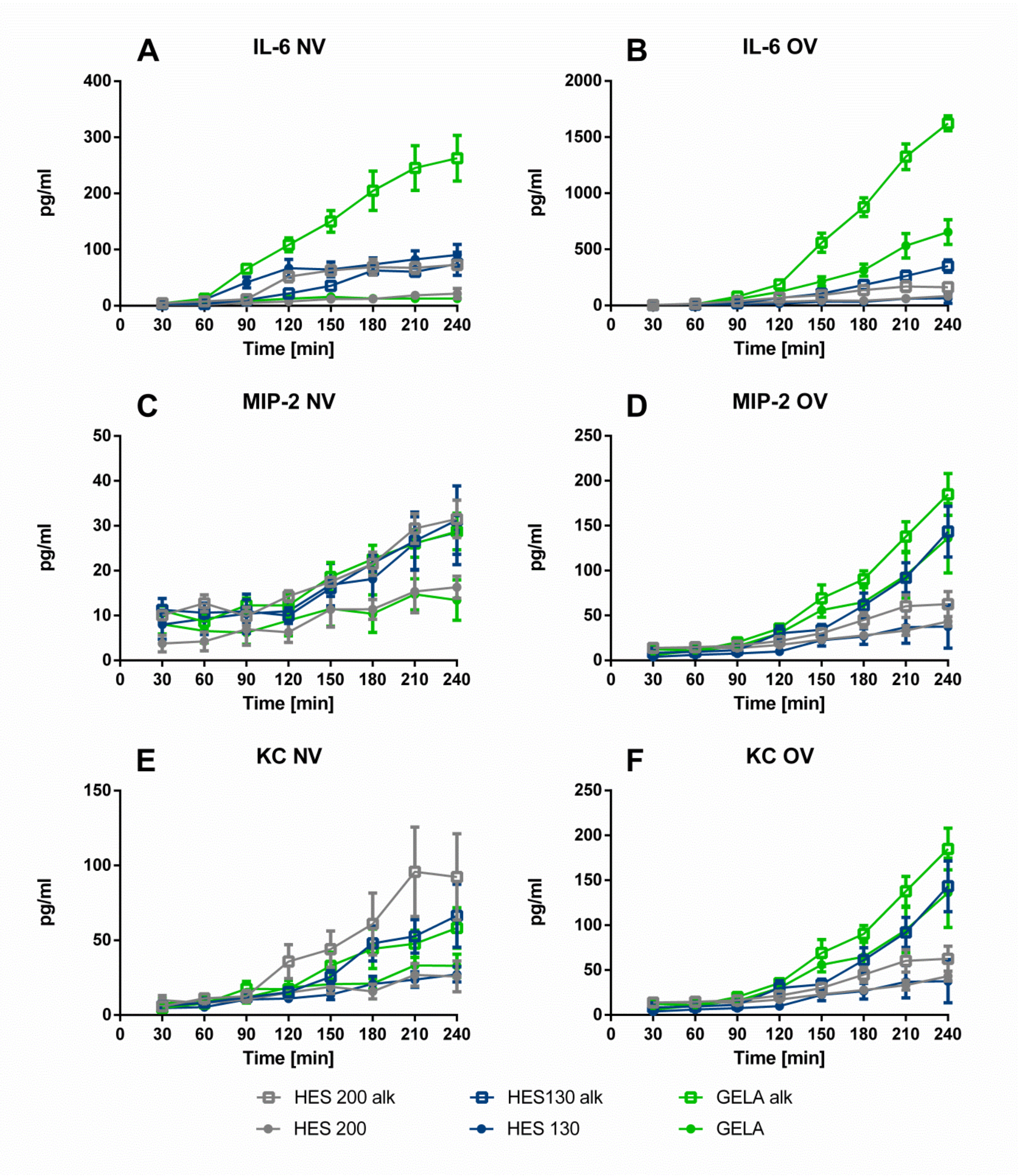

Supplemental Fig. 1: Influence of different buffers on cytokine levels: ***IL-6, MIP-2 and KC - cytokine release of isolated perfused lungs into the perfusate over 240 minutes of perfusion and ventilation. All measured cytokine levels increase significantly over 240 minutes.***

A: IL-6 levels in NV groups under normal pH and alkalosis (mean ± SEM), **B**: IL-6 levels in OV groups under normal pH and alkalosis (mean ± SEM), **C**: MIP-2 levels in NV groups under normal pH and alkalosis (mean ± SEM), **D**: MIP-2 levels in OV groups under normal pH and alkalosis (mean ± SEM), **E**: KC levels in NV groups under normal pH and alkalosis (mean ± SEM), **F**: KC levels in OV groups under normal pH and alkalosis (mean ± SEM) NV groups: HES 200 n = 6, HES 130 n = 5, GELA n = 6, HES 200 alk = 5, HES 130 alk = 5, GELA alk = 5; OV groups: HES 200: n = 5, HES 130 n = 5, GELA n = 6. HES 200 alk = 6, HES130 alk = 5, GELA alk = 5.

All depicted cytokine levels increased over time determined by univariate tests were performed using general mixed model analysis (Proc Glimmix; SAS software v9.4) assuming a lognormal distribution for all cytokine data. In case of heteroscedasticity (according to the covtest statement), the df were adjusted by the Kenward-Rogers method. Since the interaction term ‘plasma expander * timepoint’ was always significant with $p < 0.05$ an increase of all cytokine levels over time can be assumed.
